# The Impact of Housing on the Characteristics of Ceramic Pressure Sensors—An Issue of Design for Manufacturability

**DOI:** 10.3390/s151229866

**Published:** 2015-12-14

**Authors:** Marina Santo Zarnik, Darko Belavic, Franc Novak

**Affiliations:** 1 Hyb, d.o.o., Levicnikova 34, Sentjernej 8340, Slovenia; marina.santo@ijs.si; 2 HIPOT-RR d.o.o., Sentpeter 18, Otocec 8222, Slovenia; darko.belavic@ijs.si; 3 Jozef Stefan Institute, Jamova cesta 39, Ljubljana 1000, Slovenia

**Keywords:** LTCC, pressure-sensor, wet-wet application, residual stresses, finite-element analysis

## Abstract

An exploratory study of the impact of housing on the characteristics of a low-temperature co-fired ceramic (LTCC) pressure sensor is presented. The ceramic sensor structure is sealed in a plastic housing. This may have non-negligible effect on the final characteristics and should be considered in the early design phase. The manufacturability issue mainly concerning the selection of available housing and the most appropriate materials was considered with respect to different requirements for low and high pressure ranges of operation. Numerical predictions showed the trends and helped reveal the critical design parameters. Proper selection of the adhesive material remains an essential issue. Curing of the epoxy adhesive may introduce non-negligible residual stresses, which considerably influence the sensor’s characteristics.

## 1. Introduction

The integration of a sensor into a housing requires close collaboration of design engineers and manufacturing engineers in order to rationalize both the product and production. Minimizing the number of parts, adaptation to the existing housing and using the existing tooling are important steps in a design for manufacturing (DFM) process that need to be considered in the early design phase. The well-known Rule of 10 says that the cost of design defects increases tenfold with each subsequent phase of product life, from inception to obsolescence [1]. Furthermore, it is generally accepted that decisions made during the design period determine 70% of the product’s costs, which indicates the great impact that DFM can have on the product’s success on the market [2]. So far, the impact of the housing on the characteristics of ceramic pressure sensors (CPSs) has been often underestimated and mostly ignored in the early design stage. The review of the product life cycle of a pressure sensor mounted in a plastic housing exposed the need to carefully evaluate the impact of packaging on sensor characteristics. The results of an exploratory case study are presented in this paper.

Ceramic pressure sensors (CPSs) are commonly used for measurements of the pressure of various fluids and they often operate in harsh environments. For such applications it is necessary to protect the functional thick-film structures and the readout electronics from direct contact with the pressure media. This can be achieved by enclosing the sensor in an appropriate housing. Like in less robust MEMS devices [3,4], the design of housing is an important DFM issue. In order to reduce production cost it often happens that some existing housing is reused and somehow adopted. In the presented case some adjustment parts with adhesive layers are introduced to accommodate a proper sensor position in the housing.

The sensor considered in this paper is a differential pressure sensor designed for wet/wet applications. It was manufactured through the use of low-temperature co-fired ceramic (LTCC) materials and technology [3,4,5,6]. LTCC features good mechanical stability and chemical resistance together with excellent possibilities for manufacturing 3D structures with channels, cavities and thin diaphragms co-fired with thick-film sensing elements. Many articles discuss its suitability for pressure sensors [7,8,9]. Recently, an overview has been published [10]. In our previous studies the LTCC-based sensor was also tested and proved to be appropriate for use in humid environments and even in water [11,12]. Although LTCC technology inherently supports dense packaging, the interface to the LTCC module is realized by using other materials/technologies depending on the application. In this context we decided to use an existing plastic housing in order to cut down on device cost. Furthermore, since the fabrication and assembly processes are well documented through previous use, fewer defects and increased quality of the new products can be expected. However, the applied packaging solution may impact the sensor characteristics. A clear understanding of the influence of the package is therefore crucial to ensure the required performance of the designed device. An exploratory study of the impact of packaging on sensor characteristics is presented in this paper.

## 2. Experimental

### 2.1. Differential Pressure Sensor for Wet-Wet Application

Differential pressure sensors (DPS) normally operate in direct contact with the pressure media on both pressure ports. When the medium is a liquid it is a wet-wet application. The sensor discussed in this paper was designed for measurement of the water pressure. Although the LTCC sensing structure itself can be compatible with this pressure media, the electric connections and the readout electronic must be protected from it. Therefore, similarly to micro-machined MEMS sensors, the relatively robust ceramic pressure sensor is enclosed in the housings having adequate pressure ports for the measured pressure media.

**Figure 1:**
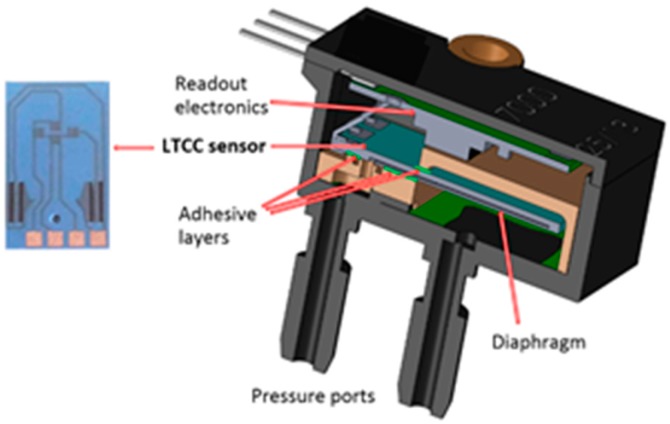
Schematic presentation of the cross section of the LTCC sensor in the housing for differential pressure measurements in a wet-wet application.

The heart of the DPS considered in this paper is an LTCC pressure sensor in the Wheatstone configuration (Figure 1), which is a slightly modified successor of the sensors that have been proven appropriate for measurements of the air pressure in water-rich environments [12]. For wet-wet measurements the functional thick-film resistors are covered with a thin protective film or are buried in an LTCC structure with the additional 50 µm LTCC layer on the top surface. The LTCC sensor is glued in an appropriate plastic housing where it is fixed in a cantilever form. The selected plastic housing serves as an alternative to a more expensive metal housing. A special two-part construction of the housing enables direct contact of the sensing diaphragm with the pressure media, while the electric connections and readout electronics parts can be protected from it. Such a structure provides good mechanical isolation of the sensing diaphragm from the stresses that can originate from the housing. However, the residual stresses introduced in the structure when curing the adhesive may affect the sensor characteristics.

We investigated possible adverse impact of packaging on the characteristics of LTCC pressure sensors. The main goal of our study was to find an appropriate adhesive material and to optimize the technological procedure. We analyzed to what extent the residual stresses introduced into the sensor structure while curing the adhesive critically affect sensor’s key performances. Numerical predictions were used to show the trends and help explain the effects of the individual material/process parameters.

### 2.2. Envisioned Problems

Initially we used finite-element analyses (FEA) to study the influence of the housing on the characteristics of the low-pressure LTCC sensors (for the pressure range up to 100 mbar) where silicone adhesive can be used and continued with specific requirements for high pressure sensors (up to 10 bar). In the latter case, epoxy-based adhesives, which will be able to withstand the stresses expected in the overload regime, are more appropriate. The epoxy adhesives have relatively high Young’s modulus (of about 3 GPa) and coefficients of the thermal expansion (CTE) > 70 × 10^−6^ K^−1^, so that significant residual stresses can remain after curing at elevated temperature of 150 °C. The curing-induced residual stresses may also enhance the internal stresses in the sensor structure under the pressure loads and so adversely influence the sensor’s burst pressure. For the low-pressure sensors, our analysis showed that in spite of the optimized construction (with good stress isolation of the sensing diaphragm), curing at 150 °C may contribute to non-negligible residual stresses influencing the offset and the temperature stability of the sensors’ characteristics. In this respect, proper selection of the adhesive material remains an essential issue. The simulations (*i.e.*, thermal-structural analyses) indirectly revealed the critical points in the manufacturing process and helped in design and process optimisation in the early prototyping phase.

Selection of appropriate adhesives depends on its compatibility with the pressure media and the desired pressure range. Preliminary tests with different commercial adhesives showed that the epoxy adhesives can be appropriate for use in the aggressive media. On the other hand, the silicone based adhesives are more appropriate for non-aggressive media and low pressure ranges. For this case study we selected two heat-curable adhesives (denoted A and B). Adhesive A was a one-component silicone adhesive and B was a one-component aluminium-powder-filled epoxy adhesive. The tensile strength (σ_max_) of adhesive A is 2.9 MPa and Young’s modulus (Y) is < 1 MPa while σ_max_ and Y of adhesive B are 66 MPa and 3.1 GPa respectively. Both materials are normally cured at 150 °C. Already from these figures it can be anticipated that the effect of curing of such different adhesives on the sensor characteristics can vary greatly. Furthermore, the residual stresses also depend on the whole structure geometry and the sensors’ pressure range.

### 2.3. Finite Element Modelling

**Figure 2:**
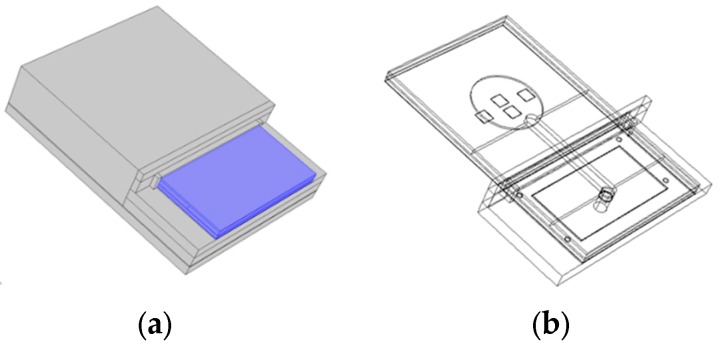
The simplified model geometry: (**a**) LTCC sensor placed in the inner part of the sensor-in-housing assembly with visible sensor part (colored blue); (**b**) wireframe view.

The model for thermal-structural analysis of the sensor-in-package and evaluation of the curing-induced residual stresses was implemented in the Comsol Multiphysics code. It was aimed at prediction of the thermal stress developed in the sensor structure and the effect of the residual stress on the key sensors characteristics: the offset voltage (*V_off_*) and the sensitivity (S). In order to reduce the model size, we modelled only the inner part of the sensor-in-housing assembly *i.e.*, the LTCC sensor fixed and glued in the plastic holder, which is glued on the bottom of the housing. The simplified geometry is presented in Figure 2. The visible part of the LTCC sensor structure, which is glued to the housing with a thin layer of adhesive, is colored blue. At this stage we neglected 10 µm conductive lines and the connection pads of the thick-film resistors, since they do not significantly affect the sensitivity, while in this way the number of required elements can be significantly reduced. As will be shown below, the analysis for LTCC sensor part (without housing) with the conductive paths was also carried out and showed very small deviation.

The key modelling assumptions for the prediction of the residual stresses induced by adhesive during cure were as follows:

Since the Young’s modulus of LTCC is significantly higher than those of the materials used for the housing and the adhesives (for at least two orders of magnitude), the formulation of the cure kinetics can be simplified. In this regard, we neglected the adhesive rheology and the effect of changes in its transient behaviors from the gel phase to the elastic solid. We assumed the final stage of the curing process at 150 °C where the adhesive is fully cured and its material properties are those specified in the manufacturer’s data sheets. A similar assumption has been made in [13] and proved to be appropriate to study the trends of the residual stresses resulting from the curing procedure.

According to the above assumptions, there are no thermal stresses in the sensor structure at the initial temperature of 150 °C. Cooling to the room temperature results in the induced residual stresses.

The material parameters used in the model were obtained from the available data sheets. For modelling thick-film piezo-resistors we built the elastoresistive coupling matrix by using the gauge coefficients evaluated in [14]. The longitudinal and transverse gauge coefficients are 11.6 and 11.4 respectively. The temperature dependence of resistivity (TCR) and the Joule heating of the thick-film resistors were not considered in this case study. We were primarily focused on the effect of the material CTEs mismatch and the resulting stresses after curing the adhesive at the elevated temperature. The CTEs of LTCC, the thick-film resistor material and the material for the housing (glass fibre reinforced semi-crystalline resin) were 5.8 × 10^−6^ K^−1^, 8.6 × 10^−6^ K^−1^, 250 × 10^−6^ K^−1^, respectively. For adhesive A we used CTE of 200 × 10^−6^ K^−1^ and for adhesive B a pricewise linear approximation, *i.e.*, CTE of 70 × 10^−6^ K^−1^, 100 × 10^−6^ K^−1^ and 186 × 10^−6^ K^−1^ in the temperature ranges 0–100, 100–125 and 125–185 °C respectively.

The effect of the residual-stress and the strain in the thick-film sensing resistor regions, resulting in changes in *V*_off_ and S, were evaluated as follows: The sensing thick-film resistors are connected in the Wheatstone’s bridge, so that the resistance changes are directly reflected in the bridge output voltage (*V*_out_). Initially all the resistors have the same initial value and *V*_out_ = *V*_off_ = 0. Due to the non-uniform residual stresses distribution and consequently different strains at the locations of the thick-film piezo-resistors, the resistance changes of the four bridge resistors are different and *V*_off_ becomes non-zero. Since we were interested in the resistance changes, rather than the actual resistances, the time-consuming coupled-field FE analysis with calculations of current density within the resistance regions was not necessary. Consequently we used a simplified approach based on structural analysis (solid mechanics interface under the Structural Mechanics branch in Comsol Multiphysics 4.4) and calculation of the resistance changes and the resulting bridge voltage in post processing. The resistance changes were obtained from the average strain components in the piezoresistive regions multiplied with the appropriate gauge coefficients. In this way we have reduced the model size and the time of calculation.

**Figure 3:**
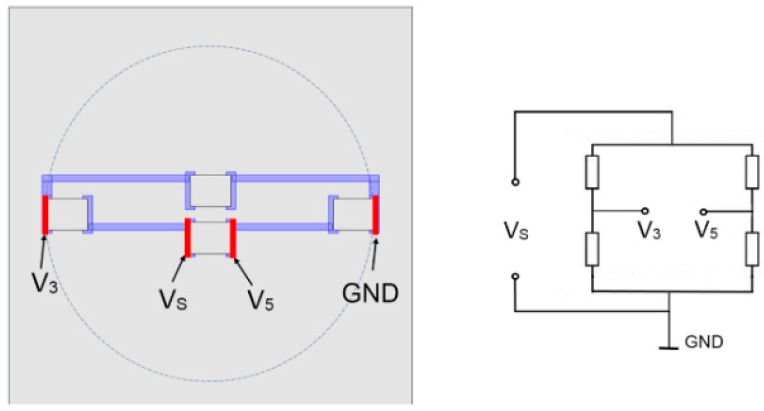
The detail of the model geometry: the LTCC sensor structure with the thick-film resistors (on the membrane) connected in the Wheatstone bridge with the metal connection paths and the electric scheme.

For verification of this simplified modelling approach we also implemented the model by using the Comsol’s Piezo-resistivity physics interface. (This was done solely for the LTCC sensor structure, without the housing.) The electric potential node and the ground nodes determining the bridge voltage (*Vs* of 5 V) were defined as the boundary condition on the appropriate resistors connection pads. The bridge output voltage was calculated as the difference between two boundary probes *V*_3_ and *V*_5_ defined as the potential on the surface of the resistor connection pads (Figure 3).

**Figure 4:**
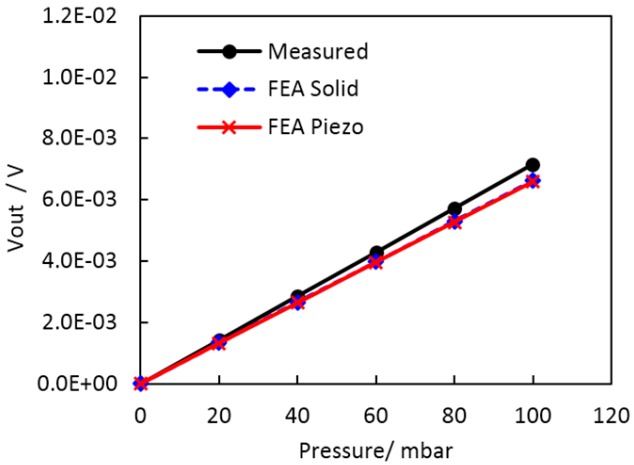
Simulated sensor output versus pressure (simplified model, denoted as FEA Solid, and piezo-resistive domain analysis, denoted as FEA Piezo) and measured characteristics of the 100-mbar sensor with a glued metal tube at 25 °C.

For validation of the model of the 100-mbar LTCC sensor (without the housing) the FEA predictions were compared to the measured response. The predicted sensitivity of 1.36 × 10^−5^ V/V/mbar (the span of 6.84 mV at the *V_S_* of 5 V) was slightly lower, but still in relatively good agreement with the typical characteristic of the sensors studied in our previous work [12,15]. The simulated and the measured output voltage versus pressure at the room temperature are presented in Figure 4.

**Figure 5:**
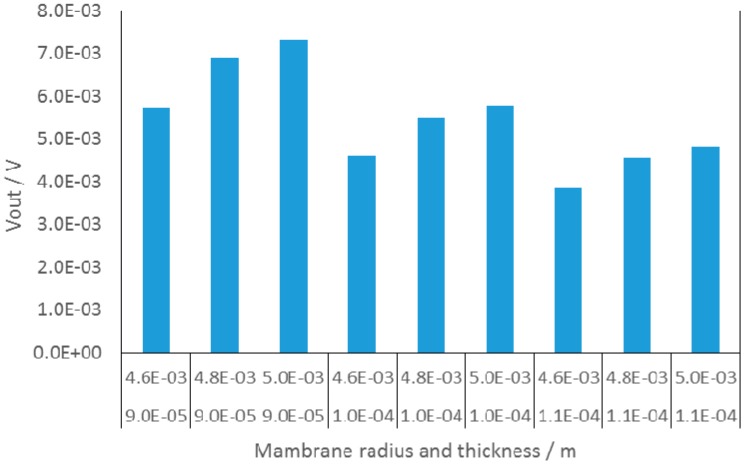
Results of parametric analysis of 100 mbar sensor.

Taking into account that we specified in the model the nominal thickness of the diaphragm, which can differ from the thickness of the processed LTCC structure (up to 10%) and that bonding of the metal tube for application of the pressure in the case of the measured prototypes can also affect the sensor response, the resulting discrepancy of the simulated and measured response was acceptable. In addition we performed further parametric analysis considering deviations of the membrane dimensions. The sensor output voltage obtained for three different membrane diameters and three different thicknesses is shown in Figure 5. It is evident that the measured data presented in Figure 4 fall within the predicted tolerance range.

The above results validated the proposed model and allowed us to perform further analysis of the LTCC sensor glued in the housing with different adhesive materials. The following cases were studied:

1. *V_off_* and the response of the 0.1-bar sensor in the temperature range −25 °C to 125 °C;
2. Changes in *V_off_* and the sensitivity of 10-bar sensors induced by adhesive curing;
3. The residual von Mises stresses and burst pressure.

## 3. Results and Discussion

### 3.1. Case of the Low-Pressure Sensor

**Figure 6:**
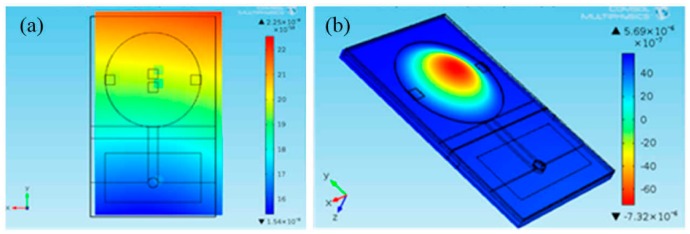
FEA predictions: (**a**) Simulated TCE-mismatch-induced displacement of the unloaded LTCC sensor structure glued in the housing by using adhesive A; (**b**) the sensor at 100 mbar pressure load.

**Figure 7:**
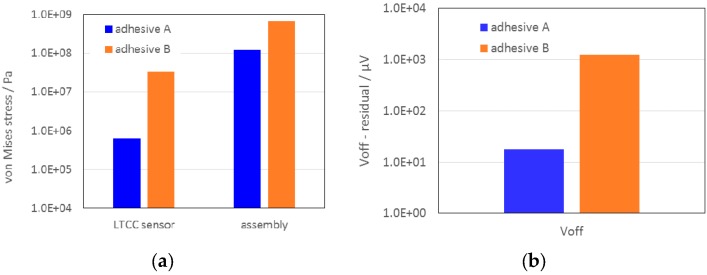
Results of FEA of the 100-mbar LTCC sensor glued with adhesive A and B: (**a**) maximum von Mises stresses in the LTCC structure; (**b**) the resulting offset voltage.

In the case of the low-pressure sensor, the simulations revealed non-negligible residual stresses remaining in the LTCC sensor structure after curing the adhesive at 150 °C. The results obtained for the 100-mbar sensor glued in the housing by using different adhesives are presented in Figure 6 and Figure 7. Figure 6a shows a rough illustration of the predicted z-component of the displacement field in the unloaded LTCC sensor structure glued in the housing by using adhesive A, while Figure 6b shows the displacement field of the sensor under the pressure load of 100 mbar.

Figure 7 shows the maximum residual von Mises stresses in the LTCC structure and the resulting *V*_off_ of the sensor glued in the housing with the selected adhesives A and B. It is evident that in the case when adhesive B is used, the residual stresses can exceed the flexural strength of LTCC. Consequently the epoxy adhesive is not suitable for the low-pressure sensors.

**Figure 8:**
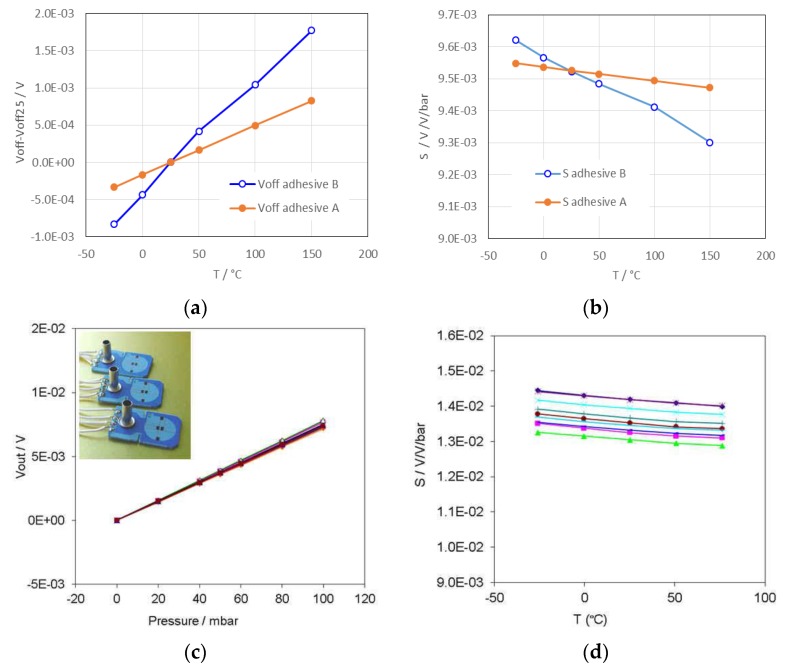
Predicted temperature characteristics of the 100-mbar sensor: (**a**) offset voltage at *V_s_* of 5 V; (**b**) sensitivity (S). Measured characteristics of 100-mbar sensors with the tubes manufactured in our previous work: (**c**) Sensors output voltage (*V_out_*); (**d**) temperature dependence of sensitivity (S).

Further FEA revealed the influence of the adhesive on the temperature dependence of the 100-mbar sensors’ characteristics. As is evident from Figure 8, the temperature dependence of both *V*_off_ and the sensitivity is significantly more evident in the case of the sensor glued with the epoxide-based adhesive B, which has higher Young’s modulus and lower CTE in comparison to the silicone-adhesive A.

The predicted temperature dependence of *V_off_* in the temperature range −25 °C to 125 °C was nearly linear, with a slope of 1.4 × 10^−5^ VK^−1^ for adhesive B and of 7.5 × 10^−6^ VK^−1^ for adhesive A. The typical temperature dependence of *V_off_*, measured for the sensors of the same type without the housing and with a metal tube for pressure inlet was about 20 × 10^−6^ VK^−1^. Similar trends have been observed in the case of 100-mbar sensors with the tubes, which we have investigated in our previous work. Measured characteristics are presented in Figure 8c,d. Although the sensitivity of this series was slightly higher the trends in temperature dependence of the sensitivity of the sensors with tubes are similar as in the case of sensors glued in the housing by silicone adhesive.

Though the simulation results somewhat differ from the above experimental data, the trends are quite similar. The differences are mainly due to the simplifications of the model, the effects of the metal tube glued on the real samples and the neglected temperature dependence of the thick-film resistors (TCR), which was not considered in the model.

For information, the temperature coefficient of sensitivity (TCS = (ΔS/S_25_)/ΔT) measured in our previous studies for the sensors with the tubes was typically about 3 × 10^−4^ K^−1^. The TCSs calculated from the simulated characteristics (in Figure 8b) are 5 × 10^−5^ K^−1^ and 2 × 10^−4^ K^−1^ for the case of the adhesive A and B respectively. Although in this case, our model is considerably simplified, the result reminds us that temperature dependence of sensitivity due to the impact of the housing should be considered in the early design phase.

### 3.2. Medium- and High-Pressure Sensors

**Figure 9:**
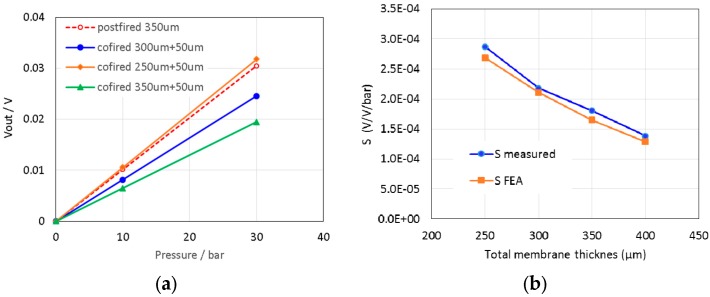
(**a**) Simulated characteristics of 10-bar sensor (at 25 °C) in dependence of the total thickness of the diaphragm; (**b**) Numerically obtained and the measured sensitivity.

Initially, the characteristics of the sensor without the housing were simulated at 25 °C for three different thicknesses of the diaphragm (Figure 9). The numerical predictions have revealed the extent to which co-firing with the 50-µm protective LTCC layer reduces the sensor’s sensitivity (Figure 9a). The characteristics were compared to the measurements of the physical samples (Figure 9b). The simulated sensitivity was slightly lower (presumably, due to the default material parameters, and partly due to systematic deviations from the nominal values of the diaphragm thicknesses that were used in the model). Such a satisfactory agreement of the experimental and the numerical results validated the model for this case study.

**Figure 10:**
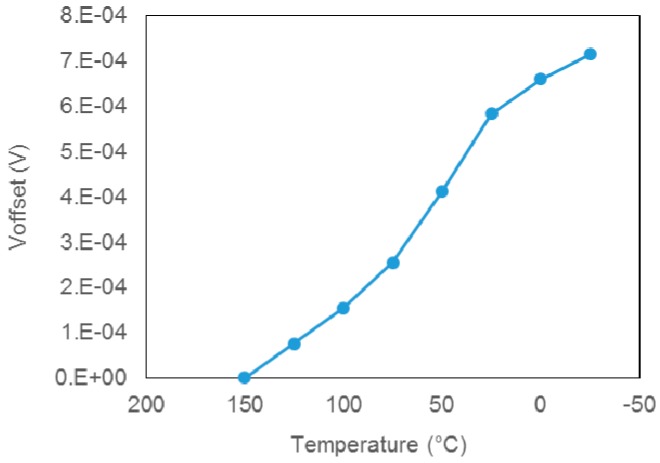
The simulated changes in *V_off_* of the 10-bar sensor (with a 350 µm membrane with the diameter of 5 mm) after curing at 150 °C and then cooling down to room temperature.

**Figure 11:**
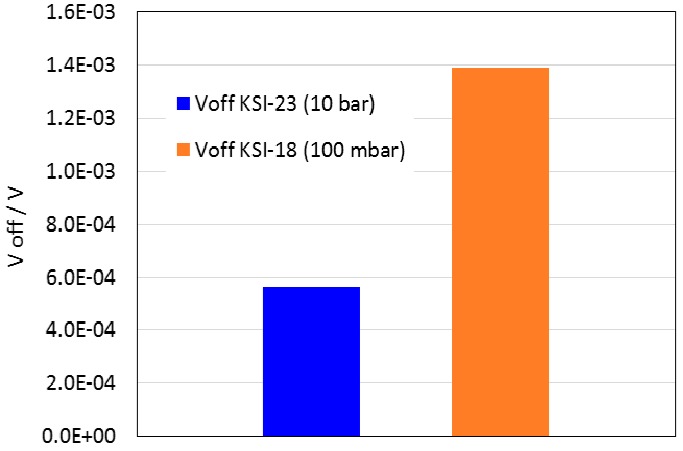
Comparison of the changes in *V_off_* induced by gluing the LTCC sensor in the housing by using adhesive B simulated for the 100-mbar and the 10-bar sensor.

In the next step, the influence of the residual stresses induced by gluing the sensor in the housing were analyzed. In this case we dealt only with adhesive B, since the analyses of the low pressure-sensor already showed that the adhesive A could not be appropriate for the higher pressure loads. In particular, we studied changes in *V_off_* and the sensor’s response to pressure loads: the full-scale pressure (FSP) of 10 bar and the designed nominal burst pressure of 25 bar. The FEA predictions (Figure 10) revealed that the residual-stress in the case of the 350 µm membrane with a diameter of 5 mm after the curing at 150 °C and then cooling down to room temperature induce a change in *V_off_* up to 600 µV. If such changes are unacceptable for the application and cannot be compensated in further production steps, the influence of the housing should be planned in the early design phase of the LTCC sensor structure. In our case the readout electronics with a dedicated commercial ASIC is used for compensation of the offset voltage, so that the discussed changes in *V_off_* are in principle not problematic, especially if there is sufficient reserve in sensitivity. However, even though in this particular case the compensation of *V_off_* of 10-bar sensor is feasible, it may be a problem for 100-mbar sensors glued with the adhesive B (Figure 11).

**Figure 12:**
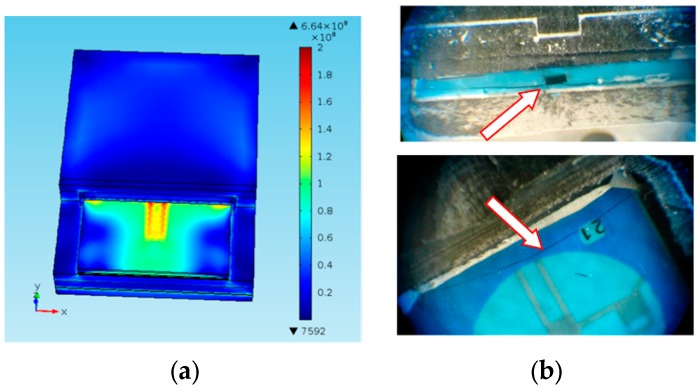
FEA-predicted distribution of the residual von Mises stress in the LTCC sensor after curing and the examples of process induced cracks in the LTCC structure. (**a**) Von Mises stress in the LTCC sensor after curing the epoxy adhesive B at 150 °C; (**b**) The cracks induced in the LTCC structure after gluing in the housing.

In all cases the simulations revealed the maximum stresses in the region of the bonding area, and particularly at the edges of the channel in the LTCC structure (Figure 12). Since these stresses can be already in the range of the flexural strength of LTCC this was the weakest point of the design. In this way, the simulations had drawn our attention to the critical points and helped us to explain some failures/defects that occurred during the manufacturing process or later during the test. For example, Figure 12b shows the process induced cracks in a non-optimized LTCC structure. The cracks appear in the critical regions at the edge of the channel (the cross section in the figure above) and along the joint with the housing (bottom).

### 3.3. Burst Pressure

One of the critical parameters when designing the pressure sensors is burst pressure, which typically should be about three times full-scale pressure (FSP). In general, for low-pressure sensors, this ratio should be higher (typically at least 5 times FSP), while for the higher-pressure ones it can be lower (typically 3 times FSP). In any case, the LTCC sensor structure must be designed so that the maximum mechanical stress appearing in the sensor structure under the loads below the burst pressure remains sufficiently lower than the flexural strength of the materials used. At the same time it is necessary to ensure sufficiently high output signal for further calibration and signal processing. In the following we present the case of 10-bar sensor for which a burst pressure of 25 bar was considered.

**Figure 13:**
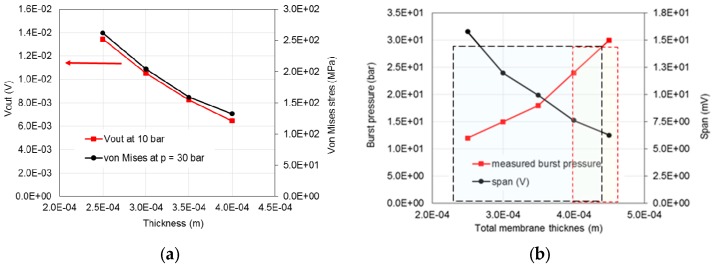
(**a**) The simulated characteristics of the sensor (at 25 °C) dependent on the total thickness of the diaphragm; (**b**) simulated span and measured burst pressure.

Simulations of the sensor structures with a diaphragm diameter of 5 mm for three different thicknesses of the diaphragm (Figure 13) showed the relation between the trends for the sensitivity of the sensor and the maximum von Mises stresses at a pressure load of 30 bar.

## 4. Conclusions

Although the ceramic pressure sensors are much more robust than the MEMS sensors, bonding into the housing, which is essential for wet-wet applications, can significantly affect their characteristics. FEA predictions for 0.1 bar and 10 bar LTCC pressure sensors sealed in the special plastic housings showed the trends and helped in revealing the effects of the individual material/process parameters in real situations. It was shown numerically and confirmed experimentally that for low-pressure applications, the silicone-based adhesive can be more appropriate then the epoxy one. If silicone adhesive is used, the changes in the sensor offset voltage are not critical, while the application of the epoxy-based adhesive can result in considerable reasonable parameter changes.

In the case of 0.1 bar sensors sealed with silicone adhesive, the CTE’s mismatch can have an observable, but still manageable influence on the temperature dependence of the sensor,s offset voltage and the sensitivity. Due to the several times lower Young’s modulus of elasticity of the silicone-based adhesive (assumed 1 MPa), its curing has almost negligible influence on the sensor’s sensitivity. All the deviations of the sensor characteristics arising after sealing in the housing can be compensated for with the commercial ASICSs for piezoresistive sensors. 

In the case of 10 bar sensors, which due to the high stresses have to be sealed with epoxy adhesive, FEA showed the residual-stresses caused an offset voltage of about 600 µV. Such an offset change can be critical because the available readout electronics can successfully compensate for the offset up to several hundred micro volts. Furthermore, the use of epoxy-based adhesives may be critical because of high residual stresses (in the range of flexural strength of LTCC) which can provoke formation and propagation of cracks in the regions with stress concentrations. Simulations revealed the origin of the failures during the manufacturing prototypes in the early optimizing phase. 

All the presented results show that the design phase of the CPS aimed at wet-wet applications must necessarily involve simultaneous optimization of the whole package (geometry and materials) and the process of gluing the sensors in the housing. The developed simulation-based approach allows us to foresee the impact of gluing the sensor part onto the housing and to assess the appropriateness of off-the-shelf housings.
